# Late-onset fabry disease due to the p.Phe113Leu variant: the first italian cluster of five families

**DOI:** 10.1007/s11011-023-01216-4

**Published:** 2023-04-25

**Authors:** Vittoria Cianci, Angelo Pascarella, Lucia Manzo, Sara Gasparini, Oreste Marsico, Anna Mammì, Carmelo Massimiliano Rao, Claudio Franzutti, Umberto Aguglia, Edoardo Ferlazzo

**Affiliations:** 1 Regional Epilepsy Centre, “Bianchi-Melacrino-Morelli” Great Metropolitan Hospital, Reggio Calabria, Italy; 2 Department of Medical and Surgical Sciences, Magna Graecia University of Catanzaro, Catanzaro, Italy; 3 Cardiology Unit, “Bianchi-Melacrino-Morelli” Great Metropolitan Hospital, Reggio Calabria, Italy; 4 Radiology Unit, “Bianchi-Melacrino-Morelli” Great Metropolitan Hospital, Reggio Calabria, Italy

**Keywords:** Late-onset Fabry Disease, p.Phe113Leu (p.F113L) pathogenic variant, Stroke, Cardiac phenotype, Neurological phenotype

## Abstract

**Background:**

The *GLA* c.337T > C (p.Phe113Leu) is a known pathogenic variant associated to late-onset Fabry disease phenotype with predominant cardiac manifestations. A founder effect was demonstrated in a large cohort in the Portuguese region of Guimarães. Herein we report an in-depth phenotype description of a cluster of five Southern Italy families.

**Methods:**

Family pedigrees of five index males with the p.Phe113Leu variant were obtained and all at-risk relatives underwent biochemical and genetical screening test. Carriers of *GLA* p.Phe113Leu variant underwent subsequent multidisciplinary clinical and instrumental evaluation.

**Results:**

Thirty-one (16 M, 15 F) individuals with p.Phe113Leu pathogenic variant were identified. Sixteen out of 31 patients (51.6%) had cardiac manifestations. Notably, myocardial fibrosis was found in 7/8 patients, of whom 2 were under 40 years. Stroke occurred in 4 patients. White matter lesions were detected in 12/19 patients and occurred in 2/10 of subjects under 40 years. Seven females complained of acroparesthesias. Renal involvement occurred in 10 patients. Angiokeratomas were evident in 9 subjects. Eyes, ear, gastrointestinal and pulmonary involvement occurred in the minority of subjects.

**Conclusion:**

This study demonstrates that a cluster of subjects with p.Phe113Leu pathogenic variant is also present in Southern Italy. Disease manifestations are frequent in both sexes and may occur early in life. Cardiac involvement represents the core manifestation, but neurological and renal involvement is also frequent, suggesting that extra-cardiac complications deserve clinical attention.

## Introduction

Fabry disease (FD; OMIM #301,500) is a rare X-linked inherited lysosomal storage disorder resulting from absent or markedly reduced activity of the α-galactosidase A (α-Gal-A) enzyme due to mutations in the *GLA* gene (OMIM #300,644) (Germain 2010). The α-Gal A enzymatic deficiency leads to globotriaosylceramides and its deacylated derivative globotriaosylsphingosine (Lyso-Gb3) lysosomal accumulation within tissue of multiple organs including heart, kidney, central and peripheral nervous systems, skin, eyes and gastrointestinal system (Germain 2010; Ortiz et al. 2018). Fabry disease usually affects hemizygous males; however, heterozygous females may exhibit variable phenotype due to the *GLA* variant or to X-chromosome inactivation profiles in various organs (Echevarria et al. 2016; Germain et al. 2022). Absent or highly reduced (< 1%) α-Gal-A activity determines the “classic” phenotype, with onset in childhood or adolescence and severe multi-systemic involvement (Germain 2010; Germain et al. 2022). Higher residual enzymatic activity leads to milder single organ (mainly heart or kidney) forms of the disease with late onset (“atypical” or “late-onset” FD, LOFD; Germain 2010; Ortiz et al. 2018; Wilcox et al. 2008). Currently, more than 1000 *GLA* different mutations have been identified (http://www.dbfgp.org/dbFgp/fabry; http://www.fabrygenphen.com).

The *GLA* missense c.337T > C (p.Phe113Leu ) is a known pathogenic variant, associated with late-onset disease Azevedo et al. 2020a, b; Oliveira et al. 2020). It was firstly identified in an adult male with mild cardiac-variant FD (Eng et al. 1997) and subsequently described in other case reports and population screening studies (Burlina et al. 2008; Favalli et al. 2016; Hagège et al. 2011; Lee et al. 2010; Nowak et al. 2018; Park et al. 2009; Spada et al. 2006; Veroux et al. 2020; Vigneau et al. 2021). Recently, this pathogenic variant was found in a great number of families in the Portuguese region of Guimaraes and a founder effect has been demonstrated (Azevedo et al. 2020b). In all reported cases, disease phenotype was predominantly cardiac Azevedo et al. 2020a, b; Hagège et al. 2011; Oliveira et al. 2020); however, renal, central and peripheral nervous system involvement as well as sensorineural deafness have been reported Azevedo et al. 2020a, b; Burlina et al. 2008; Favalli et al. 2016; Oliveira et al. 2020; Veroux et al. 2020; Vigneau et al. 2021).

Herein we provide an in-depth phenotype description of five Italian families living in Southern Italy, with LOFD caused by p.Phe113Leu pathogenic variant.

## Materials and methods

### Subjects

**Fig. 1:**
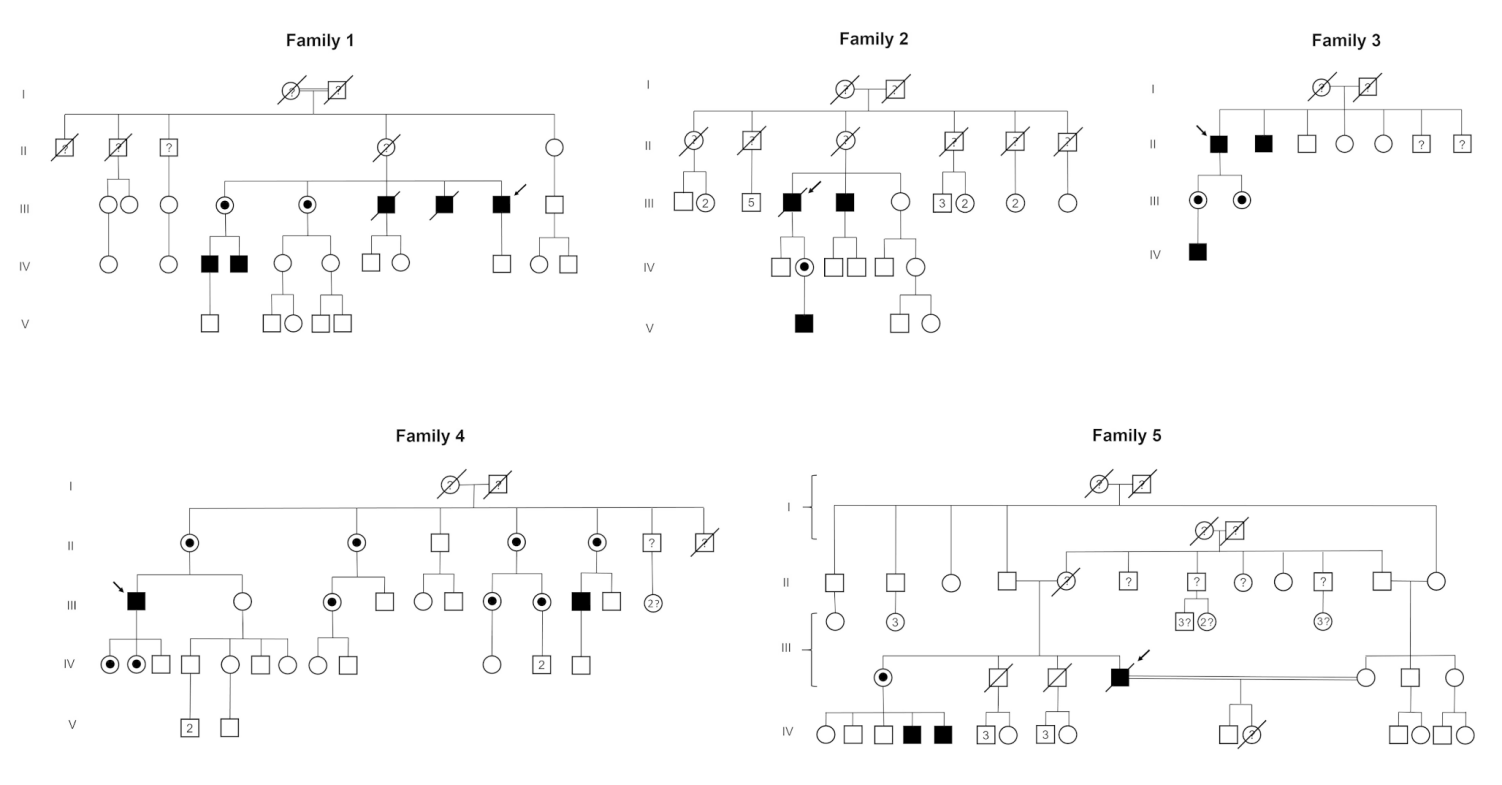
Family pedigrees from the five patients with *GLA* p.Phe113Leu variant Index patients are marked with a black arrow. Numbers within symbols denote number of male or female relatives; question mark denotes subjects unavailable for biochemical and genetic evaluation due to refusal or death

Four men with symptomatic left ventricular hypertrophy (LVH) and one man with ischemic stroke occurred at age 58 years, underwent screening test for FD (α-Gal-A enzymatic activity and plasmatic Lyso-Gb3 levels) with subsequent identification of p.Phe113Leu variant in *GLA* gene in all of them. They referred to the Rare Disease Center of our hospital and received complete clinical evaluation. Family pedigree was obtained for all the five probands (Fig. 1). All at-risk relatives were invited to undergo screening test (Gal et al. 2011; Michaud et al. 2020; Stiles et al. 2020) for FD (α-Gal-A enzymatic activity for males, plasmatic Lyso-Gb3 level and genetic study for both males and females). Subjects identified as carriers of *GLA* pathogenic variant underwent subsequent detailed clinical evaluation.

### Biochemical and genetic evaluation

Enzymatic activity of α-Gal-A was measured trough dried blood spot technique (Chamoles et al. 2001), using high performance liquid chromatography (HPLC) and tandem mass. Normal value of α-Gal-A enzymatic activity and plasmatic Lyso-Gb3 were > 15.3 µ/l/h and < 1.8 ng/ml, respectively. Genetic study was achieved by DNA isolation from whole blood and application of DNA next-generation sequencing (NGS) of the target region (NM_000169.2).

### Clinical and instrumental evaluation

The predefined protocol comprised multidisciplinary evaluation. Cardiac evaluation comprised standard ECG or Holter-ECG monitoring, echocardiography, cardiac magnetic resonance imaging (C-MRI). Diagnosis of LVH was defined as the presence of LV wall thickness ≥ 15 mm (Azevedo et al. 2021). Neurological investigation included brain MRI (or brain CT, when MRI was unavailable), color-doppler ultrasound (or angio-TC) of carotid arteries, electroneurography when clinically indicated. Nephrological evaluation included blood and urine analysis (serum creatinine, proteinuria, albuminuria on 24 h-urine and albumin/creatinine ratio on random urine, estimation of glomerular filtration rate (eGFR) by chronic kidney disease-epidemiology collaboration (CKD-EPI) formula) and kidney ultrasound (if indicated). The protocol also included ophthalmologic, dermatologic, gastroenterological, pneumological, psychological/psychiatric and otorhinolaryngology examination, with instrumental evaluation when needed. Symptomatic patients underwent 6 months follow-up evaluation, while asymptomatic individuals without relevant alterations at clinical and instrumental evaluations were followed yearly.

## Results

Screening of at risk relatives of the probands resulted in identification of 31 individuals (16 M, 15 F) with *GLA* p.Phe113Leu variant (Fig. 1). Main demographic, clinical and biochemical features of patients are summarized in Table 1. Mean age at diagnosis was 53.8 years both in male and female. Of the 31 subjects, 7 were members of family I, 4 of family II, 5 of family III, 11 of family IV and 4 of family V. Seventeen of the 31 patients fulfilled diagnostic criteria for late-onset FD diagnosis, while the other 14 were asymptomatic carriers. The α-Gal-A activity levels were < 2.8 μm/l/h in all tested subjects. Plasmatic Lyso-Gb3 levels were available for 29 patients and pathological values were found in 19 (65.5%) subjects. Sixteen (51.6%) patients manifested cardiac involvement (Table 1). Cardiac imaging was performed in 20 patients. Left ventricular hypertrophy was found in 12 (60%) subjects, with slight predominance in males (6/16 M and 6/15 F); all males but one were over 40-year-old. Diastolic dysfunction occurred in 6 (30%) subjects. Eight patients performed C-MRI and late gadolinium enhancement (LGE) was revealed in 7 (87.5%); three of them had concomitant LVH. Moreover, 2 out of 7 subjects with LGE were under 40 years. History of myocardial infarction was reported in 2 males. Heart conduction disturbances were found in 7 (22.5%) subjects, leading to the implantation of a pacemaker in 3 of them.

**Table 1:** Clinical phenotype of subjects with *GLA* p. Phe113Leu variant. α-GAL A: α-galactosidase A; IVS: interventricular septum; LGE: late gadolinium enhancement; LV: left ventricular; LVH: left ventricular hypertrophy; Lyso-Gb3: globotriaosylsphingosine ; PW: posterior wall.; WML: white matter lesions

Demographical and clinical characteristics				All patients (n: 31)	Male (n: 16)	Female (n: 15)
Age at diagnosis (years; mean ± SD)				53.8 ± 19.1	53.8 ± 19.9	53.8 ± 19.0
Biochemical markers						
	Low plasma α-GAL A activity (< 2.8 nmol/h/mL)^1^			16 (100%)	16 (100%)	-
	Mean plasma Lyso-Gb3 (ng/mL)^2^			3.1 ± 2.6	6.3 ± 1.7	1.4 ± 0.4
Cardiac manifestations				16 (51.6%)	8 (50%)	8 (53.3%)
	Myocardial ischemic events (n, %)			2 (6.4%)	2 (12.5%)	0
	Heart failure (n, %)			7 (22.5%)	5 (31.2%)	2 (13.3%)
	Cardiac imaging^3^					
		LVH		12 (60%)	6 (66.7%)	6 (54.5%)
			IVS thickness (mm; mean ± SD)	13.9 ± 4.8	15.0 ± 1.4	13.75 ± 5.2
			PW thickness (mm; mean ± SD)	10.2 ± 2.0	9.0 ± 1.3	10.4 ± 2.1
			LV mass (g/m^2^; mean ± SD)	127.1 ± 62.2	133.0 ± 42.4	125.2 ± 70.9
		LV ejection fraction (%; mean ± SD)		61.8 ± 8.3	58.3 ± 11.9	62.8 ± 7.4
		LV diastolic dysfunction (n, %)		6 (30%)	1 (11.1%)	5 (45.4%)
	LGE (n, %)^4^			7 (87.5%)	2 (100%)	5 (83.3%)
	Arrhythmia (n, %)			7 (22.5%)	3 (18.7%)	4 (26.7%)
	Pacemaker (n, %)			3 (9.6%)	2 (12.5%)	1 (6.7%)
Neurological and neuropsychiatric manifestations				14 (45.1%)	5 (31.2%)	9 (60%)
	Stroke (n, %)			4 (12.9%)	3 (18.7%)	1 (6.7%)
	WML (n, %)^5^			12 (63.1%)	4 (57.1%)	8 (66.6%)
	Brain haemorrhage (n, %)^5^			1 (5.2%)	1 (14.3%)	0
	Acroparesthesias (n, %)			7 (22.5%)	0	7 (46.7%)
	Carpal tunnel syndrome (n, %)^6^			3 (15%)	0	3 (20%)
	Polyneuropathy (n, %)^6^			2 (10%)	1 (14.3%)	1 (7.7%)
	Dysautonomia (n, %)			6 (19.3%)	3 (18.7%)	3 (20%)
	Anxiety/depression (n, %)			3 (9.6%)	0	3 (20%)
Renal manifestations				10 (32.2%)	4 (25%)	6 (40%)
	Albuminuria (n, %)			6 (19.3%)	2 (12.5%)	4 (26.7%)
	Proteinuria (n, %)			4 (12.9%)	2 (12.5%)	2 (13.3%)
	Kidney insufficiency (n, %)			3 (9.6%)	0	3 (20%)
Skin Manifestation						
	Angiokeratomas (n, %)			9 (29%)	2 (12.5%)	7 (46.7%)
Eye manifestation				5 (16.1%)	1 (6.2%)	4 (26.6%)
	Cornea verticillata (n, %)			1 (3.2%)	0	1 (6.7%)
	Cataracts (n, %)			3 (9.6%)	1 (6.2%)	2 (13.3%)
	Retinal vessel tortuosity (n, %)			1 (3.2%)	0	1 (6.7%)
Ear manifestations						
	Sensorineural deafness (n, %)			3 (9.6%)	0	3 (20%)
Pulmonary manifestations (n, %)				2 (6.4%)	1 (6.25%)	1 (6.7%)
Gastrointestinal manifestations (n, %)				4 (12.9%)	1 (6.25.%)	3 (20%)
Comorbidity						
	Hypertension (n, %)			9 (29%)	3 (18.7%)	6 (40%)
	Diabetes mellitus (n, %)			4 (12.9%)	2 (12.5%)	2 (13.3%)

^**1**^Performed in 16/31; ^2^performed in 29/31 (16 M and 13 F); ^3^cardiac imaging performed in 20/31 (9 M and 11 F); ^4^cardiac-MRI performed in 8/31 patients (2 M and 6 F); ^5^brain imaging performed in 19/31 patients (7 M and 12 F); ^6^ nerve conductions study performed in 20/31 patients (7 M and 13 F)

Stroke occurred in 4 patients (age range 58–70 years). Brain imaging was performed in 19 patients. Singular, multiple or confluent T2-weighted hyperintense MRI lesions (white matter lesions, WML) were detected in 12 (63.1%, Table 1) subjects and represented an early finding, as they were found in 2/5 (40%) of the subjects under 40 years. Interestingly, 4/12 patients with WML had no cerebrovascular risk factors. Peripheral nervous system (PNS) involvement was found in 5 subjects (Table 1). In addition, autonomic dysfunction (hypohidrosis, dysfunctional gastrointestinal motility) was found in 6 patients. Seven females complained with acroparesthesias. Neuropsychiatric manifestations (i.e. depression, anxiety disorder) occurred in 3 subjects.

Renal involvement (albuminuria and/or proteinuria) was found in 10 patients (Table 1); 3/10 were younger than 40 years. Renal insufficiency was rare: stage 1 chronic kidney failure was evident only in 3 females (all older than 60 years). Angiokeratomas were found in 9 (29%) patients. Ocular and hearing manifestations were uncommon (Table 1).

## Discussion

This study demonstrated the presence of a FD cluster due to *GLA* c.337T > C (p.Phe113Leu) pathogenic variant in Southern Italy. The *GLA* c.337T > C mutation is known to be responsible for late-onset cardiac phenotype since it was found in patients with predominantly cardiac manifestations Azevedo et al. 2020a, b, 2021; Eng et al. 1997; Hagège et al. 2011; Oliveira et al. 2020). Clinical, biochemical and instrumental data confirmed that *GLA* c.337T > C (p.Phe113Leu) pathogenic variant results in a LOFD with a predominant cardiac phenotype. Clinical or instrumental signs of cardiac involvement were found in about two thirds of subjects. The main cardiac abnormality was LVH, which was found in 60% of our patients and in 40.4% of a large cohort of Portuguese FD subjects (Azevedo et al. 2020b). These results seem to confirm a higher prevalence of LVH in *GLA* c.337T > C variant as compared to other variants with predominant cardiac involvement such as IVS4 and p.N215S (Hsu et al. 2016; Lavalle et al. 2018). C-MRI has been demonstrated to be a valuable tool for an early assessment of cardiac fibrosis through demonstration of LGE (Perry et al. 2019). Studies providing C-MRI data on cardiac predominant FD phenotypes are scant. We found LGE in 7 out of 8 of our subjects, thus confirming the usefulness of C-MRI. Although an age-dependent incidence of LGE is known (Aquaro et al. 2022), we found LGE in two patients aged under 40 years, suggesting that myocardial fibrosis can occur early in p.Phe113Leu FD variant. Moreover, LVH was found only in 3 patients with LGE, suggesting that LGE may precede LVH (Niemann et al. 2011). In agreement with Azevedo et al. (2020b), hearth failure developed in about a quarter of our patients, whereas myocardial ischemia was less frequent and occurred exclusively in two males. Occurrence of heart arrhythmias is common in classic as well as late-onset variant FD (Azevedo et al. 2021; Hagège et al. 2019; Linhart et al. 2007; Pieroni et al. 2021). In the p.Phe113Leu variant, rhythm and conduction disorders, including atrial fibrillation and flutter, atrioventricular block, fascicular block and non-sustained ventricular tachycardia, have been described Azevedo et al. 2020a, b; Oliveira et al. 2020). Accordingly, cardiac rhythm disturbances were frequent in our cohort too, occurring in 22.5% of subjects, with necessity of pacemaker implantation in 9.6% of subjects.

Central or peripheral nervous system involvement was demonstrated in half of our patients. Stroke is a common and important manifestation in both “classic” and “late-onset” variant FD (Arends et al. 2017; Buechner et al. 2008; Cianci et al. 2022; Brouns et al. 2010; Sims et al. 2009), including prevalent cardiac FD phenotype Alharbi et al. 2018; Azevedo et al. 2020b; Germain DP et al.2018; Lee et al. 2016, 2017). Stroke represented the first FD manifestation in one of our index cases and occurred in 12.9% of whole cohort, mainly in male. A lower incidence of stroke (3.0%) was found in Azevedo et al. series (Azevedo et al. 2020a); this may be explained by differences in the two population’s characteristics, such as higher mean age at stroke and smaller sample of our study.WML occurred in 63.1% of our subjects and were evident also in young patients. Our findings are in line with previous reports Azevedo et al. 2020a, b) and confirm that a small vessel brain disease represents a common finding of FD and may arise early in the course of disease (Kolodny et al. 2015). PNS involvement frequently occur in FD (Cianci et al. 2022; Ortiz et al. 2018; Ranieri et al. 2016). We found acroparesthesias in nearly half of our patients and carpal tunnel syndrome exclusively in one woman. Our findings confirm the female predominance of acroparesthesias and carpal tunnel syndrome previously observed Azevedo et al. 2020a, b; Oliveira et al. 2020).

We found kidney involvement in about a third of our patients. However, the main signs of renal disease were increased albuminuria or proteinuria, whereas low grade renal insufficiency was a rare event. No subject in our series underwent renal dialysis or transplant. Kidney involvement has been reported in p.Phe113Leu variant Azevedo et al. 2020b, b; Burlina et al. 2008; Favalli et al. 2016; Oliveira et al. 2020; Veroux et al. 2020; Vigneau et al. 2021) as well as other prevalently cardiac LOFD (Di Stefano et al. 2021; Germain et al. 2018; Lavalle et al. 2018), rarely requiring renal dialysis or transplant (Arends et al. 2017; Germain et al. 2018; Veroux et al. 2020; Vigneau et al. 2021). Our findings confirms that kidney involvement is often mild and slowly progressive in the p.Phe113Leu LOFD variant Azevedo et al. 2020a, b). Interestingly, it still remains to be elucidated whether kidney involvement in FD due to p.Phe113Leu variant is due to the disease itself or to the presence of other risk factors such as age, hypertension or diabetes mellitus (Azevedo et al. 2020b; Oliveira et al. 2020; Smirnova et al. 2020). In our cohort, albuminuria/proteinuria was also found in subjects under 40 years and none of these patients had major additional risk factors. Our findings corroborate the hypothesis that FD itself may play a major role in kidney disease pathogenesis.

## Conclusions

In conclusion, we provide a description of FD phenotype due to the *GLA* p.Phe113Leu pathogenic variant in a large cohort of subjects belonging to five Italian families. Our findings confirm that the *GLA* missense c.337T > C (p.Phe113Leu) variant causes a LOFD with a predominant cardiac phenotype. Central and peripheral nervous system as well as kidney involvement may often occur even if the cause-effect relationship with the *GLA* p.Phe113Leu variant remains to be fully elucidated.

## Data Availability

The datasets generated during and/or analysed during the current study are available from the corresponding author on reasonable request.
